# A Chromosomal Microarray Detects Microdeletion at Chromosome Locus 11p14.3-p12 Leading to Wilms Tumor, Aniridia, Genitourinary Anomalies, and Mental Retardation (WAGR) Syndrome

**DOI:** 10.7759/cureus.72479

**Published:** 2024-10-27

**Authors:** Renuka A Majjigudda, Pramila Menon, Supriya Gupte, Vishesh Dikshit, Vishwanath Kulkarni, Shailaja Mane, Parag M Tamhankar

**Affiliations:** 1 Pediatrics, Dr. D. Y. Patil Medical College, Hospital and Research Centre, Dr. D. Y. Patil Vidyapeeth (Deemed to be University), Pune, IND; 2 Pediatric Endocrinology, Dr. D. Y. Patil Medical College, Hospital and Research Centre, Dr. D. Y. Patil Vidyapeeth (Deemed to be University), Pune, IND; 3 Pediatric Surgery, Deenanath Mangeshkar Hospital and Research Centre, Pune, IND; 4 Pediatric Neurology, Dr. D. Y. Patil Medical College, Hospital and Research Centre, Dr. D. Y. Patil Vidyapeeth (Deemed to be University), Pune, IND; 5 Genetics, Dr. D. Y. Patil Medical College, Hospital and Research Centre, Dr. D. Y. Patil Vidyapeeth (Deemed to be University), Pune, IND

**Keywords:** aniridia, microdeletion, pax6, penile hypospadias, snp microarray, wagr syndrome, wilms tumor, wt1

## Abstract

The short form of the term "WAGR syndrome" denotes susceptibility to Wilms tumor, absence of irises, genital and urinary anomalies, and growth/development retardation. It is also called 11p deletion syndrome since varying amounts of the short arm of chromosome 11 are found deleted in these patients. The earliest presenting symptom can be undescended testes detected at birth or nystagmus, which can bring attention to the aniridia by a physician. Recognition of this disorder is important for surveilling Wilms tumor, an embryonal cancer of the kidney. A genetic diagnosis is possible by using a chromosomal microarray, fluorescent in situ hybridization, or multiplex ligation-dependent probe amplification (MLPA). The inheritance is autosomal dominant and, in most cases, the deletion is sporadic/denovo (not inherited from parents). We describe a male child with Wilm's tumor, aniridia, genitourinary anomalies, and mental retardation (WAGR) syndrome due to a microdeletion on chromosome 11 {arr[GRCh38]11p14.3p12(22,560,576_38,466,045)x1}.

## Introduction

Wilm's tumor, aniridia, genitourinary anomalies, and mental retardation (WAGR) syndrome is a contiguous gene deletion syndrome characterized by a *denovo* interstitial deletion on the p arm of chromosome 11, including the *WT1* and *PAX6* genes. Miller, Fraumeni, and Manning first recognized it in 1964 and described it as an association between Wilms tumor, absence of irises, hemihypertrophy, and genital and urinary tract abnormalities [1]. The G in WAGR refers to growth anomalies, ambiguous genitals, gonadoblastoma, or genital/urinary tract abnormalities. Riccardi et al. and Anderson et al., in the year 1978, showed that the mechanism is due to an interstitial deletion [2,3]. The deleted segment common to most patients is the distal half of 11p13. The aniridia is due to *PAX6* gene deletion, whereas Wilms tumor predisposition is due to *WT1* gene deletion. Some patients show obesity, wherein the syndrome is denoted as WAGRO. Obesity is due to hyperphagia caused by the deletion of the *BDNF* gene (brain-derived neurotrophic factor) [4]. A subset of patients is denoted as AGR syndrome (without Wilms tumor) due to deletion of region 11p14.1-p13. The frequency of this disease is one in 5-10 lakh individuals. A genetic diagnosis can be made possible by using a chromosomal microarray, fluorescent in situ hybridization (FISH), or multiplex ligation-dependent probe amplification (MLPA) [5]. We describe a male child with WAGR syndrome diagnosed on chromosomal microarray to have microdeletion on chromosome 11.

## Case presentation

**Figure 1:**
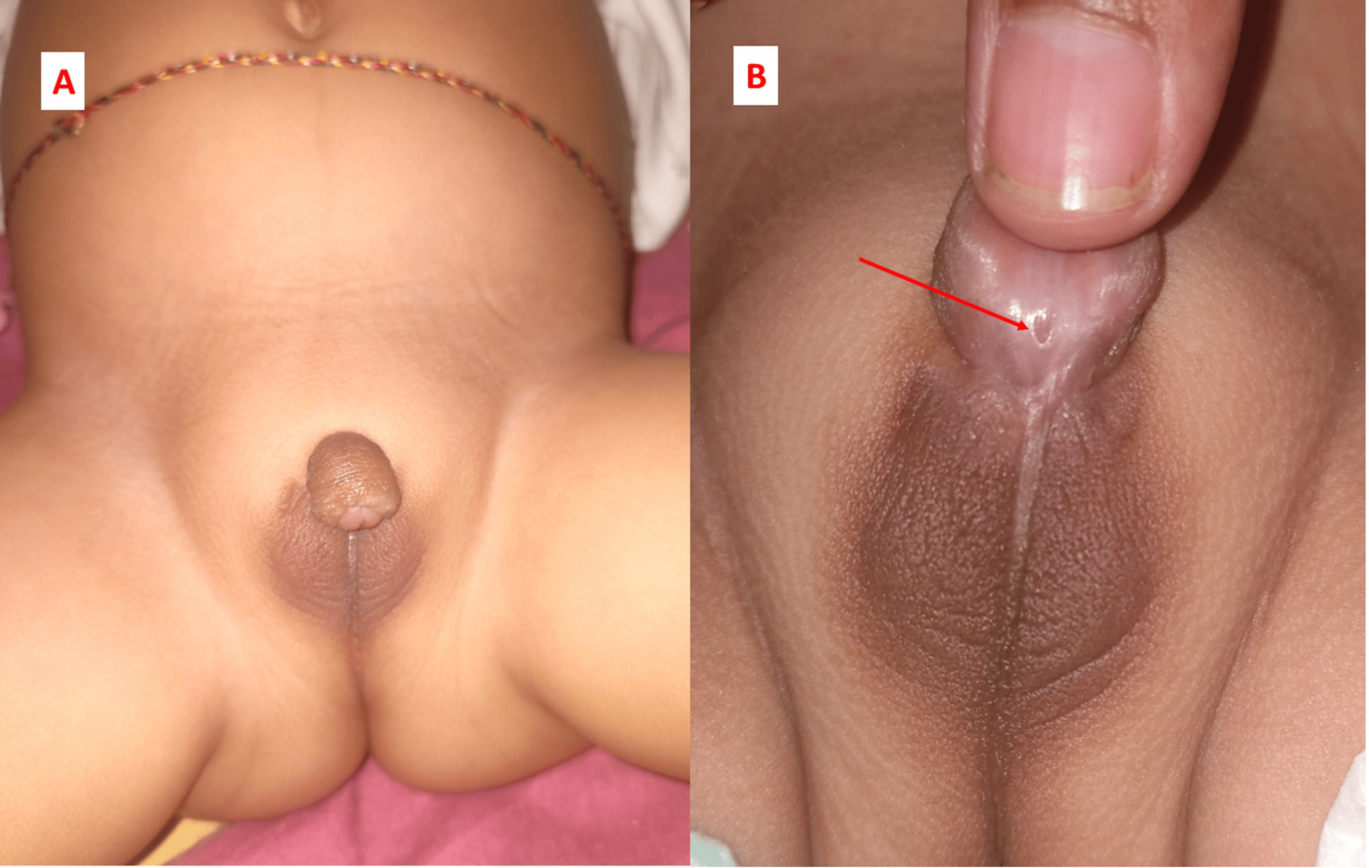
Undescended testes (A) and proximal penile hypospadias (B) 1A shows micropenis, right undescended (but palpable) testis and left undescended testis, and the scrotal sac is ill-formed with minimal rugosity. 1B shows a close-up of the testes and proximal hypospadias, and the red arrow shows the urethral opening.

The patient was first born of third-degree consanguineous parents. He was born at term, vaginally, cried well after birth, and birth weight of 2,100 g. He was first noticed at birth to have bilateral undescended testes and proximal penile hypospadias (see Figures 1A, 1B).

**Figure 2:**
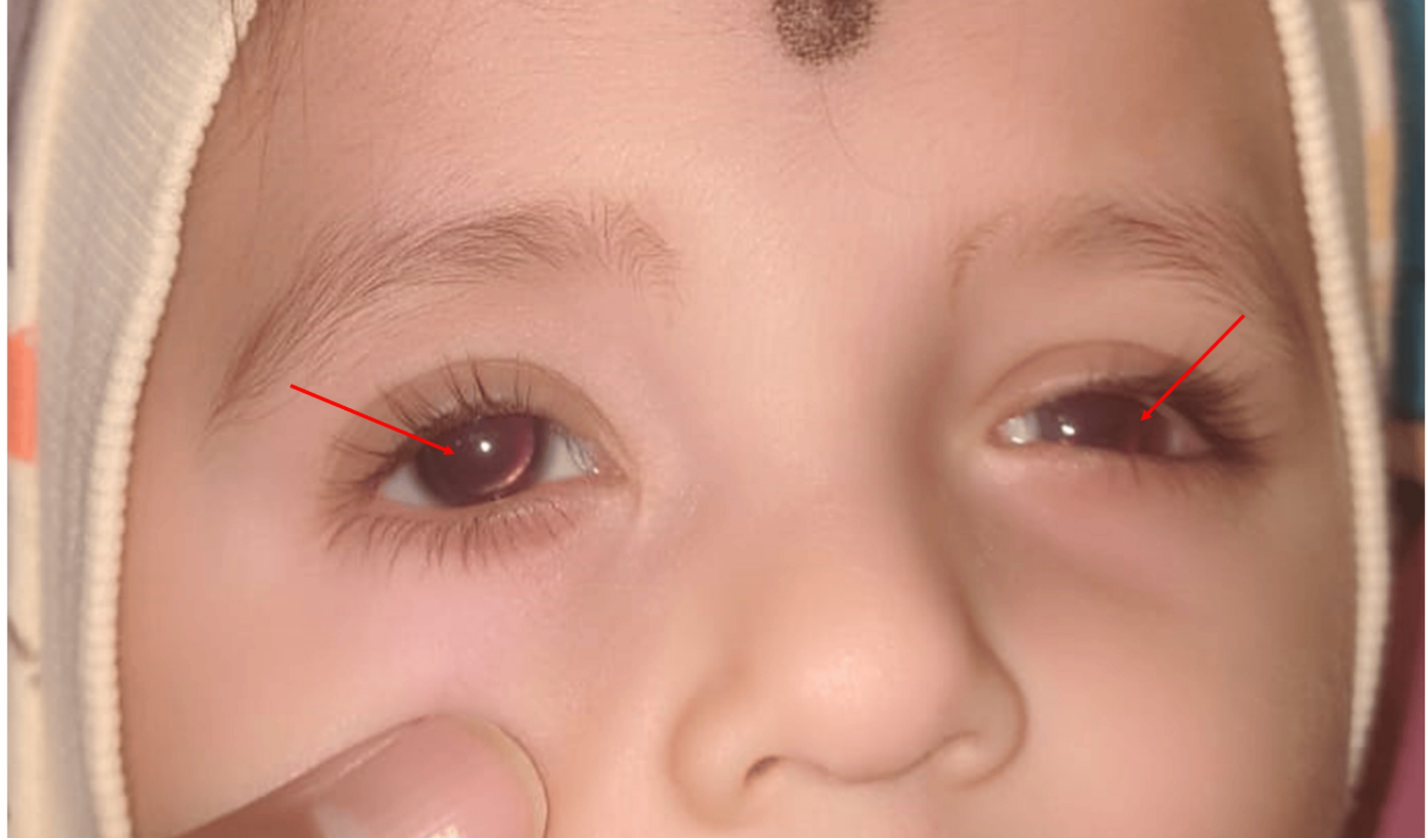
Aniridia A close-up of the eyes shows bilateral aniridia (red arrows), pupils appear dilated, the lens is clear, and no facial dysmorphism is noted.

He was told to wait by the pediatrician for six months after which surgery can be performed for the hypospadias. He was later noticed at three months of age by his parents not to have any visual contact and squeezed his eyes in response to bright light. Examination by an ophthalmologist revealed bilateral absence of irises (see Figure 2).

**Figure 3:**
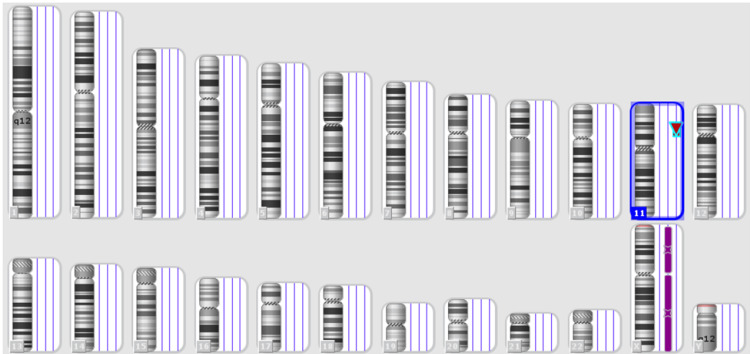
Chromosomal microarray showing microdeletion on chromosome 11 Virtual karyogram depicting ideograms of all 23 chromosomes. A heterozygous deletion is present on the short arm of chromosome 11 depicted by an inverted red triangle. The purple line next to the X chromosome indicates a loss of heterozygosity due to the presence of only a single X chromosome (the patient is male with an XY sex chromosome complement).

**Figure 4:**
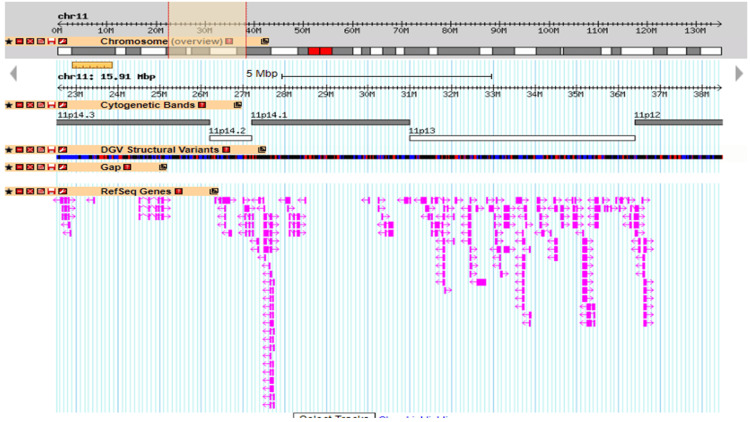
Detailed view of the chromosome 11 microdeletion on the Database of Genomic Variant website A representation of the microdeletion arr[GRCh38]11p14.3p12(22,560,576_38,466,045)x1. The topmost horizontal lane is a scale showing that chromosome 11 is made up of around 130 megabases of genomic material. The second horizontal lane is an entire chromosome 11 ideogram that shows alternate dark and light bands with a shaded region between two red vertical lines focusing on 22.5 megabase to 38.5 megabase region. The third horizontal lane is the scale with the focused region between 22.5 megabase to 38.5 megabase region. The fourth horizontal lane shows that various cytobands present in the deleted region, namely, 11p14,3, 11p14,2, 11p14.1, 11p13, and 11p12. The bottom last horizontal broad lane shows various genes colored in pink that are present in the deleted region.

They were referred to an eye institute in Hyderabad at eight months. They did a detailed eye examination. On slit lamp examination, the conjunctiva, cornea, and sclera were normal in both eyes. Anterior chambers in both eyes appeared deep. The undilated pupils were bilaterally 10 mm in size due to aniridia. The lenses were clear. Intra-ocular pressure measured by applanation tonometry was 13 mm of Hg in the right eye and 19 mm in the left eye (normal). On fundus examination, the media was clear, and optic discs were small bilaterally. The cup-disc ratio was 0.2 in both eyes. Retinal blood vessels were normal. Maculae showed absent foveal reflex in both eyes indicating poor macular development. The ophthalmologist demonstrated and explained the visual stimulation exercises to the parents to enhance the fixing and following skills of non-illuminated objects. They recommended high contrast, bright colored, simple patterned bigger toys for visual stimulation. They suggested maintaining eye level while communicating or training to elicit the child's eye contact. They advised the child to wear a peaked cap when going outside to avoid glare issues. A detailed development assessment was performed by a pediatrician around 8.5 months because of complaints of delayed development. At that age, he could roll over on both sides, put weight on his forearm in the prone position, and had partial head control. He had decreased visual contact and focus. He could be alerted to auditory stimuli. He smiled occasionally. He could put his hand to his mouth. Development assessment was repeated at 11 months. At that age, he could roll over from supine to prone and vice versa. When in the prone position, he could prop himself up on his hands but was unable to sustain it for a long time. With little assistance, he could make a transition from prone to quadruped and then into a kneeling posture. The physiotherapists were encouraging him to maintain an unaided kneeling posture with weight bearing on his hands, from there on progressing from half kneeling to standing. He could thus become able to stand with support. The other features observed at 11 months included good eye contact, lateral localization of sound, transfer of objects from one hand to the other, and vocalizing dada, baba. The physiotherapists used supra malleolar orthoses to treat his pronated feet. Various exercises prescribed were as follows: figure of four sitting, prone posture on hands, supine to sit pull, creeping, quadruped hold, prone to kneeling transition, aided push-ups, abdominal muscle strengthening on ball, crunches/obliques, sitting to standing transitions, etc. The parents were also advised to use a lot of hand and face gestures, point and name body parts and objects, and play peek-a-boo games. An ultrasound of the abdomen performed at 11 months showed the right kidney to be 55 x 35 mm in size and the left kidney to be 59 x 31 mm in size. Both kidneys showed normal position, echotexture, cortico-medullary differentiation, and pelvicalyceal system. There was no calculus or mass. The urinary bladder was normal. An ultrasound of the inguinoscrotal region at 11 months showed that the right testis was in the distal aspect of the inguinal canal and measured 15 x 6.7 x 6 mm (normal). The left testis could neither be visualized in the scrotal sac nor the abdomen. Stretched penile length was 2 cm (micropenis). Three tubular structures were noted within the penis on ultrasound, likely spongiosum and cavernosum. Both spermatic cords appeared normal. Chromosomal microarray-based cytogenetic analysis by Cytoscan 750K on the Affymetrix platform (ThermoFisher Scientific Inc., Waltham, MA) showed a heterozygous deletion on chromosome 11 at 11p14.3-p12 region of about 15,905 Kb {arr[GRCh38]11p14.3p12(22,560,576_38,466,045) x1} (see Figure 3 and Figure 4). 

The list of genes included in this region and their clinical phenotypes listed in the Online Mendelian Inheritance of Man website are listed in Table 1.

**Table 1:** List of genes present in the microdeleted region on the short arm of chromosome 11 A list of genes included in the arr[GRCh38]11p14.3p12(22,560,576_38,466,045) x1 microdeletion, with their corresponding phenotypes, inheritance models of their associated diseases, Online Mendelian Inheritance in Man (OMIM) database condition numbers, OMIM disease conditions. AD: autosomal dominant inheritance, AR: Autosomal recessive inheritance, N/A: not applicableR

Genes	Dosage Senstivity	Matching Phenotypes	Inheritance Model	OMIM Condition Numbers	OMIM Conditions
WT1	Yes	Aniridia, Neoplasm, Renal neoplasm, Aplasia/Hypoplasia of the iris, Abnormality of the nervous system, Nephroblastoma, Hypospadias	Somatic Mutation, AD	194080, 194070, 136680, 256370, 608978, 156240	Denys-Drash syndrome, Wilms tumor 1, Frasier syndrome, nephrotic syndrome, type 4, Meacham syndrome, malignant mesothelioma
PAX6	Yes	Aniridia, Neurodevelopmental delay, Renal neoplasm, Aplasia/Hypoplasia of the iris, Motor delay, Nephroblastoma, Hypospadias, Delayed gross motor development, Abnormality of the eye, Global developmental delay	N/A	106210, 165550, 148190, 604229, 136520, 120200, 120430	aniridia 1, isolated optic nerve hypoplasia, autosomal dominant keratitis, Peters anomaly, foveal hypoplasia 1, coloboma, ocular, autosomal dominant, coloboma of optic nerve
CAT	N/A	N/A	AR, Unknown	614097	acatalasia
CCDC34	N/A	N/A	AR	620084	spermatogenic failure 76
CAPRIN1	N/A	Global developmental delay	N/A	620782, 620636	neurodevelopmental disorder with language impairment, autism, and attention deficit-hyperactivity disorder, neurodegeneration, childhood-onset, with cerebellar ataxia and cognitive decline
BDNF	Yes	Aniridia, Renal neoplasm, Aplasia/Hypoplasia of the iris, Nephroblastoma, Hypospadias	AD	N/A	N/A
SLC1A2	N/A	Global developmental delay	AD	617105	developmental and epileptic encephalopathy, 41
GAS2	N/A	N/A	N/A	N/A	N/A
FANCF	N/A	Neoplasm, Aplasia/Hypoplasia of the iris, Hypospadias, Abnormality of the urinary system, Abnormality of the eye, Global developmental delay	AR	603467	Fanconi anemia complementation group F
PDHX	N/A	Global developmental delay, Motor delay	AR	245349	pyruvate dehydrogenase E3-binding protein deficiency
CD59	N/A	N/A	N/A	612300	primary CD59 deficiency
RAG2	N/A	N/A	N/A	603554, 601457, 233650	Omenn syndrome, severe combined immunodeficiency, autosomal recessive, T cell-negative, B cell-negative, NK cell-positive, combined immunodeficiency with skin granulomas
RAG1	N/A	N/A	AR	603554, 601457, 609889, 233650	Omenn syndrome, severe combined immunodeficiency, autosomal recessive, T cell-negative, B cell-negative, NK cell-positive, combined immunodeficiency due to partial RAG1 deficiency, combined immunodeficiency with skin granulomas
FSHB	N/A	N/A	AR	229070	hypogonadotropic hypogonadism 24 without anosmia
TRIM44	Yes	Aniridia	AD	617142	aniridia 3
ELP4	Yes	Aniridia, Abnormality iris morphology	N/A	617141	aniridia 2
KCNA4	N/A	Delayed gross motor development, Motor delay	AR	618284	microcephaly, cataracts, impaired intellectual development, and dystonia with abnormal striatum
ANO3	N/A	N/A	AD	615034	dystonia 24
LGR4	Yes	N/A	AD	619613, 615311	delayed puberty, self-limited, Bone mineral density quantitative trait locus 17
CD44	N/A	N/A	Unknown	609027	Blood Group, Indian System
LINC01616	N/A	N/A	N/A	N/A	N/A
DNAJC24	N/A	N/A	N/A	N/A	N/A
BBOX1-AS1	N/A	N/A	N/A	N/A	N/A
TRAF6	N/A	N/A	N/A	N/A	N/A
CCDC73	N/A	N/A	N/A	N/A	N/A
LOC100506675	N/A	N/A	N/A	N/A	N/A
ANO3-AS1	N/A	N/A	N/A	N/A	N/A
KIAA1549L	N/A	N/A	N/A	N/A	N/A
FJX1	N/A	N/A	N/A	N/A	N/A
EIF3M	N/A	N/A	N/A	N/A	N/A
MIR610	N/A	N/A	N/A	N/A	N/A
KIF18A	N/A	N/A	N/A	N/A	N/A
RCN1	N/A	N/A	N/A	N/A	N/A
COMMD9	N/A	N/A	N/A	N/A	N/A
NAT10	N/A	N/A	N/A	N/A	N/A
WT1-AS	N/A	N/A	N/A	N/A	N/A
CCDC179	N/A	N/A	N/A	N/A	N/A
LMO2	N/A	N/A	N/A	N/A	N/A
MIR8087	N/A	N/A	N/A	N/A	N/A
MPPED2	N/A	N/A	N/A	N/A	N/A
FBXO3	N/A	N/A	N/A	N/A	N/A
PAMR1	N/A	N/A	N/A	N/A	N/A
SLC5A12	N/A	N/A	N/A	N/A	N/A
LOC101928510	N/A	N/A	N/A	N/A	N/A
ARL14EP	N/A	N/A	N/A	N/A	N/A
MUC15	N/A	N/A	N/A	N/A	N/A
LINC00294	N/A	N/A	N/A	N/A	N/A
MIR8054	N/A	N/A	N/A	N/A	N/A
LUZP2	N/A	N/A	N/A	N/A	N/A
CSTF3	N/A	N/A	N/A	N/A	N/A
HIPK3	N/A	N/A	N/A	N/A	N/A
FIBIN	N/A	N/A	N/A	N/A	N/A
ELF5	N/A	N/A	N/A	N/A	N/A
IMMP1L	N/A	N/A	N/A	N/A	N/A
MIR1343	N/A	N/A	N/A	N/A	N/A
C11orf74	N/A	N/A	N/A	N/A	N/A
LINC02546	N/A	N/A	N/A	N/A	N/A
PRR5L	N/A	N/A	N/A	N/A	N/A
METTL15	N/A	N/A	N/A	N/A	N/A
ABTB2	N/A	N/A	N/A	N/A	N/A
CSTF3-DT	N/A	N/A	N/A	N/A	N/A
QSER1	N/A	N/A	N/A	N/A	N/A
LINC00678	N/A	N/A	N/A	N/A	N/A
BDNF-AS	N/A	N/A	N/A	N/A	N/A
LOC100507144	N/A	N/A	N/A	N/A	N/A
PRRG4	N/A	N/A	N/A	N/A	N/A
PAUPAR	N/A	N/A	N/A	N/A	N/A
SVIP	N/A	N/A	N/A	N/A	N/A
C11orf91	N/A	N/A	N/A	N/A	N/A
BBOX1	N/A	N/A	N/A	N/A	N/A
TCP11L1	N/A	N/A	N/A	N/A	N/A
LDLRAD3	N/A	N/A	N/A	N/A	N/A
DEPDC7	N/A	N/A	N/A	N/A	N/A
FBXO3-DT	N/A	N/A	N/A	N/A	N/A
SNORA88	N/A	N/A	N/A	N/A	N/A
LINC02760	N/A	N/A	N/A	N/A	N/A
LGR4-AS1	N/A	N/A	N/A	N/A	N/A
MIR8068	N/A	N/A	N/A	N/A	N/A
LIN7C	N/A	N/A	N/A	N/A	N/A
PAX6-AS1	N/A	N/A	N/A	N/A	N/A
MIR3973	N/A	N/A	N/A	N/A	N/A
DCDC1	N/A	N/A	N/A	N/A	N/A
SNORD164	N/A	N/A	N/A	N/A	N/A
EHF	N/A	N/A	N/A	N/A	N/A
APIP	N/A	N/A	N/A	N/A	N/A

Magnetic resonance imaging of the brain done at five months was normal. No family history of similar illness was present, and parents did not have aniridia. The child on present examination at 12 months of age showed horizontal nystagmus, aniridia, no other dysmorphic facial features, proximal penile hypospadias, right palpable (inguinal) undescended testis, and left non-palpable, undescended testis. His anthropometry was normal for his age. Head control was present, but he could not sit on his own. He had visual contact and could follow objects. He had a social smile but did not have any stranger anxiety. He could babble.

A human chorionic gonadotropin (HCG) stimulation test was performed at 11 months of age. The baseline values were as follows: serum follicle-stimulating hormone 1.20 mIU/mL (normal range: 0-5 mIU/mL), serum luteinizing hormone 0.83 mIU/mL (normal range: 0.04-3.6), and serum testosterone was less than 12.98 ng/dL (less than 15 ng/dL). Post HCG stimulation serum testosterone rose to 122.82 ng/dL, suggestive of a good response and the presence of functional testicular tissue. Anti-Mullerian hormone levels were more than 18 ng/mL, indicative of good Sertoli cell function (normal range: 18-283 ng/mL). Thus, hormonal evaluation was suggestive of a functional right testis.

The surgical plan of the child to increase the size of the phallus will be as follows: 1) diagnostic laparoscopy for the left nonpalpable testis, followed by either a single-stage or two-stage orchidopexy depending on the length of the gonadal vessels provided that there is confirmation of the presence of the left testis intra-abdominally; 2) single-stage orchidopexy for the right palpable undescended testis; and 3) two-stage repair for the penoscrotal hypospadias following either a) testosterone gel (1 % strength) application to the penis for 30 days before surgery or b) intramuscular injection of testosterone enanthate 2 mg/kg monthly for three months before surgery.

The patient's family underwent genetic counseling. They were explained that this is a sporadic autosomal dominant genetic disorder. Karyotyping of the child was advised to rule out chromosomal rearrangements that can predispose to a deletion. They were counseled that the child may have a visual handicap. They were explained the importance of ultrasound abdomen every three months till eight years of age, to screen for Wilms tumor. Annual intra-ocular pressure measurements were needed to detect glaucoma. Parents were also told to screen the child regularly for kidney function beginning around 10 years of age. Prenatal diagnosis possibility in further pregnancies of the couple by fetal microarray around 12-16 weeks of pregnancy was also counseled.

## Discussion

The differential diagnosis of this disorder is as follows. Isolated aniridia can be caused by point mutations in the *PAX6* gene (aniridia type 1), *ELP4* gene (chromosome 11p13) (aniridia type 2), and *TRIM44* gene (chromosome 11p13) (aniridia type 3). Aniridia can also be a part of anterior segment dysgenesis syndromes, such as the Peters anomaly and the Axenfeld-Rieger syndrome. Peters anomaly refers to the central corneal opacity. The Axenfeld anomaly refers to the prominent Schwalbe line and sticky peripheral iris strands, and the Rieger anomaly refers to iris stromal atrophy leading to the pupil being displaced from the central position or forming a hole or pseudo-hole [6]. Mutations in the *WT1* gene can also lead to Frasier syndrome or Denys-Drash syndrome [7]. The Frasier syndrome refers to ambiguity in sex or sex reversal in males, streak gonads, and chronic kidney disease due to focal segmental glomerulosclerosis. The Frasier syndrome occurs due to mutation in the *WT1* gene leading to the production of a shorter protein that lacks the KTS domain such as donor splice site mutation in intron 9. The risk of Wilms tumor is low. The Denys-Drash syndrome refers to genital ambiguity and sex reversal in males, gonadal dysgenesis, chronic kidney disease due to diffuse mesangial sclerosis, and high Wilms tumor risk. This syndrome occurs due to dominant negative mutations in the *WT1 *gene (such as missense mutations) [7].

We also briefly discuss the published literature on WAGR syndrome. Fischbach et al. published a cohort of 54 WAGR syndrome cases [8]. The age range of patients was from seven months to 42 years. Twenty-four patients had all four classical findings, 14 had three, 11 had two, and five had only one. Four of the five had aniridia with the 11p13 deletion, and one had Wilms tumor with the deletion. Wilms tumor afflicted 31 (57%) patients with 27 requiring nephrectomy and all requiring chemotherapy (actinomycin D and vincristine). Mental retardation occurred in 39 (70%) patients. Sixty percent of males had undescended testes, and 17% of females had either streak ovaries/bicornuate uterus. Genital ambiguity was present in two boys and three girls. Fourteen patients (26%) had renal failure, which could be due to bilateral nephrectomy (due to Wilms tumor) or focal segmental glomerulosclerosis. Proteinuria ranged from minimal to nephrotic range. The important nonclassical findings in the patients included cataracts (36 patients), glaucoma (24 patients), nystagmus (22 patients), optic nerve hypoplasia (8 patients), macular/foveal hypoplasia (7 patients), retinal detachment (5 patients), hypertonia/hypotonia (7 patients), epilepsy (4 patients), asthma (8 patients), need for tonsillectomy/adenoidectomy (22 patients), recurrent sinusitis (15 patients), obstructive sleep apnea (11 patients), recurrent otitis media (10 patients), attention deficit hyperactivity disorder (12 patients), autism (10 patients), tight Achilles tendon (9 patients), scoliosis/kyphosis (8 patients), and obesity (10 patients) [8]. An annual ophthalmic checkup is advised to detect glaucoma/cataract development.

Duffy et al. studied 91 patients from the WAGR syndrome patient registry enrolled between 2014 and 2020 [9]. They observed dysmorphic facial features low-set ears, down-slanting palpebral fissures, prognathism, strabismus, and amblyopia. Aniridia was present in all but two participants. Around 50% developed Wilms tumor and/or nephrogenic rests (NR). NR are clusters of immature cells of the kidney that mimic a tumor. A biopsy may be needed to differentiate from Wilms tumor. Six participants in the study developed only NR without Wilms tumor. Among those who developed Wilms tumor, more than half were diagnosed by 18 months, more than 75% were diagnosed around three years and 95% were diagnosed by age five years [9]. Children are advised to undergo three monthly ultrasounds of the kidneys till eight years of age to detect Wilms tumor. Female children with streak ovaries should also undergo regular ultrasounds to screen for gonadoblastoma.

Breslow et al. [10] reviewed 5,910 patients diagnosed with Wilms tumor between 1969 and 1994. They identified 17 patients with Denys-Drash syndrome and 37 patients with WAGR. The cumulative incidence of end-stage renal disease at 20 years after diagnosis of Wilms tumor was 74% for Denys-Drash syndrome and 36% for WAGR syndrome patients [10].

Crolla et al. [11] tested patients of aniridia for 11p13 deletion by FISH using a panel of cosmids, including *WT1, PAX6*, and flanking markers. They found a high percentage of cases with deletions (4 out of 14 familial cases (28.5%) and 26 out of 63 sporadic cases (41%)). In most families, the deletion is sporadic [11]. However, in rare cases, for chromosomal rearrangements, such as that described by Henry et al. [12], a pericentric intrachromosomal insertion can lead to the recurrence of 11p13p14 deletion in the family [12]. In rare cases, the deletion can be inherited from an affected parent [13]. In some cases, balanced translocations between chromosome 11 and other chromosomes can lead to recurrent WAGR syndrome. Examples of translocations include t(11;13) and t(11;12) [11]. The most important genes in the deleted region are *WT1* and *PAX6*, which are 700 kilobases apart. The size of deletion of 11p13 varies between 1 megabase to 26.5 megabase; however, even a 700 kilobase deletion deleting both *WT1* and *PAX6* genes can cause WAGR syndrome [14].

## Conclusions

WAGR syndrome is caused by chromosome 11p microdeletion syndrome that includes the critical genes *WT1* and *PAX6*. Inheritance is autosomal dominant, but most cases are sporadic. Surveillance for Wilms tumor is important for early detection and appropriate management. Definitive diagnosis is possible by chromosomal microarray, which can aid syndrome identification, genetic counseling, and preventing recurrences in the family by prenatal diagnosis. The inheritance is autosomal dominant, and recurrence risk in sporadic cases is low (less than 1%) but may be higher in cases with familial translocations, and up to 50% if a parent is affected. Reporting of cases increases awareness of this rare condition and promotes further research.

## References

[1] Miller RW, Fraumeni JF Jr, Manning MD (1964). Association of Wilms’s tumor with aniridia, hemihypertrophy and other congenital malformations. N Engl J Med.

[2] Riccardi VM, Sujansky E, Smith AC, Francke U (1978). Chromosomal imbalance in the aniridia-Wilms' tumor association: 11p interstitial deletion. Pediatrics.

[3] Andersen SR, Geertinger P, Larsen HW, Mikkelsen M, Parving A, Vestermark S, Warburg M (1977). Aniridia, cataract and gonadoblastoma in a mentally retarded girl with deletion of chromosome II. A clinicopathological case report. Ophthalmologica.

[4] Han JC, Liu QR, Jones M (2008). Brain-derived neurotrophic factor and obesity in the WAGR syndrome. N Engl J Med.

[5] Blanco-Kelly F, Palomares M, Vallespín E (2017). Improving molecular diagnosis of aniridia and WAGR syndrome using customized targeted array-based CGH. PLoS One.

[6] Wawrocka A, Krawczynski MR (2018). The genetics of aniridia - simple things become complicated. J Appl Genet.

[7] Lopez-Gonzalez M, Ariceta G (2024). WT1-related disorders: more than Denys-Drash syndrome. Pediatr Nephrol.

[8] Fischbach BV, Trout KL, Lewis J, Luis CA, Sika M (2005). WAGR syndrome: a clinical review of 54 cases. Pediatrics.

[9] Duffy KA, Trout KL, Gunckle JM, Krantz SM, Morris J, Kalish JM (2021). Results from the WAGR syndrome patient registry: characterization of WAGR spectrum and recommendations for care management. Front Pediatr.

[10] Breslow NE, Collins AJ, Ritchey ML, Grigoriev YA, Peterson SM, Green DM (2005). End stage renal disease in patients with Wilms tumor: results from the National Wilms Tumor Study Group and the United States Renal Data System. J Urol.

[11] Crolla JA, van Heyningen V (2002). Frequent chromosome aberrations revealed by molecular cytogenetic studies in patients with aniridia. Am J Hum Genet.

[12] Henry I, Hoovers J, Barichard F (1993). Pericentric intrachromosomal insertion responsible for recurrence of del(11)(p13p14) in a family. Genes Chromosomes Cancer.

[13] Robinson DO, Howarth RJ, Williamson KA, van Heyningen V, Beal SJ, Crolla JA (2008). Genetic analysis of chromosome 11p13 and the PAX6 gene in a series of 125 cases referred with aniridia. Am J Med Genet A.

[14] Clericuzio C, Hingorani M, Crolla JA, van Heyningen V, Verloes A (2011). Clinical utility gene card for: WAGR syndrome. Eur J Hum Genet.

